# Towards validation in clinical routine: a comparative analysis of visual MTA ratings versus the automated ratio between inferior lateral ventricle and hippocampal volumes in Alzheimer’s disease diagnosis

**DOI:** 10.1007/s00234-024-03280-8

**Published:** 2024-01-19

**Authors:** Mandy M. J. Wittens, Gert-Jan Allemeersch, Diana M. Sima, Tim Vanderhasselt, Steven Raeymaeckers, Erik Fransen, Dirk Smeets, Johan de Mey, Maria Bjerke, Sebastiaan Engelborghs

**Affiliations:** 1 Dept. of Biomedical Sciences, University of Antwerp, Universiteitsplein 1, 2610 Antwerp, Belgium; 2 Dept. of Neurology, Universitair Ziekenhuis Brussel (UZ Brussel), Av. du Laerbeek 101, 1090 Brussels, Belgium; 3 Dept. of Radiology, Universitair Ziekenhuis Brussel (UZ Brussel), Av. du Laerbeek 101, 1090 Brussels, Belgium; 4 Icometrix, Kolonel Begaultlaan 1b, 3012 Leuven, Belgium; 5 AI Supported Modelling in Clinical Sciences (AIMS), Vrije Universiteit Brussel, Pleinlaan 2, 1050 Brussels, Belgium; 6 StatUa Center for Statistics, University of Antwerp, Universiteitsplein 1, 2610 Antwerp, Belgium; 7 NEUR (Neuroprotection & Neuromodulation), Center for Neurosciences (C4N), Vrije Universiteit Brussel (VUB), Av. du Laerbeek 101, 1090 Brussels, Belgium; 8 Laboratory of Neurochemistry, Dept. of Clinical Chemistry, Universitair Ziekenhuis Brussel (UZ Brussel), Av. du Laerbeek 101, 1090 Brussels, Belgium

**Keywords:** Automated brain volumetry, Magnetic resonance imaging, Alzheimer’s disease, Medial temporal lobe atrophy, Biomarker, Dementia

## Abstract

**Purpose:**

To assess the performance of the inferior lateral ventricle (ILV) to hippocampal (Hip) volume ratio on brain MRI, for Alzheimer’s disease (AD) diagnostics, comparing it to individual automated ILV and hippocampal volumes, and visual medial temporal lobe atrophy (MTA) consensus ratings.

**Methods:**

One-hundred-twelve subjects (*mean age* ± *SD*, 66.85 ± 13.64 years) with varying degrees of cognitive decline underwent MRI using a Philips Ingenia 3T. The MTA scale by Scheltens, rated on coronal 3D T1-weighted images, was determined by three experienced radiologists, blinded to diagnosis and sex. Automated volumetry was computed by icobrain dm (v. 5.10) for total, left, right hippocampal, and ILV volumes. The ILV/Hip ratio, defined as the percentage ratio between ILV and hippocampal volumes, was calculated and compared against a normative reference population (*n* = 1903). Inter-rater agreement, association, classification accuracy, and clinical interpretability on patient level were reported.

**Results:**

Visual MTA scores showed excellent inter-rater agreement. Ordinal logistic regression and correlation analyses demonstrated robust associations between automated brain segmentations and visual MTA ratings, with the ILV/Hip ratio consistently outperforming individual hippocampal and ILV volumes. Pairwise classification accuracy showed good performance without statistically significant differences between the ILV/Hip ratio and visual MTA across disease stages, indicating potential interchangeability. Comparison to the normative population and clinical interpretability assessments showed commensurability in classifying MTA “severity” between visual MTA and ILV/Hip ratio measurements.

**Conclusion:**

The ILV/Hip ratio shows the highest correlation to visual MTA, in comparison to automated individual ILV and hippocampal volumes, offering standardized measures for diagnostic support in different stages of cognitive decline.

**Supplementary information:**

The online version contains supplementary material available at 10.1007/s00234-024-03280-8.

## Introduction

Currently, the standard diagnostic dementia work-up consists of a clinical evaluation, a full neuropsychological examination, and MR imaging of the brain, which often includes visual inspection of different brain regions using standardized rating scales, such as Scheltens’ medial temporal lobe atrophy (MTA) [41, 42] and the global cortical atrophy (GCA) scale [30]. On structural MRI, beyond mild MTA due to normal aging, at least moderate MTA is suggestive for AD, in particular when the hippocampus is affected [33, 48]. Hippocampal atrophy is an established biomarker of Alzheimer’s disease (AD) [18, 29, 47]. Co-occurrence of reduced hippocampal volumes and inferior lateral ventricle (ILV) enlargement, the two regions used to formulate the MTA score, is also typical for atrophy due to neurodegeneration in case of AD and facilitates the differentiation with individuals with congenitally small hippocampi [11, 14, 46]. However, especially in earlier symptomatic stages of AD, hippocampal atrophy is hard to objectify by visual inspection [39]. The hippocampus is a small structure which, together with the existing variability in hippocampal volumes (also present in the normal population), complicates the detection of atrophy due to AD. According to Harper et al. 2015 [13], the reliability of visual rating scales is satisfactory, with an intraclass correlation coefficient (*ICC*) of 0.8 for MTA, and 0.6 for GCA, for inter-rater reliability. However, since visual rating scales suffer from MRI acquisition protocol dependency, difficulties in identifying subtle distinctions, intra- and inter-rater variability and require trained neuroradiologists, automated volumetric assessment has become increasingly important as an additional measure for AD diagnostics, aiming to diagnose AD earlier, e.g., in the prodromal stage. The added diagnostic value of automated hippocampal volumetry to the diagnostic confidence of AD (beyond neuropsychological evaluation, cerebrospinal fluid AD biomarkers, and brain FDG-PET scan) has been shown and emphasized in previous literature [3]. Even though widely used in research settings, the integration of automated volumetry in routine clinical practice is still an ongoing evolving process [7, 16, 25]. As suggested by Vernooij et al. [51], one of the main concerns hampering integration of automated software largely pertains to lack of standardization, validation, concerns about specificity, and the difficulty to transfer research findings into the clinical setting to help diagnosing an individual patient [24]. To overcome potential shortcomings of automated hippocampal volumetry alone for (early) AD diagnosis, we suggest the use of an automated MTA ratio, defined as the ratio between ILV and hippocampal volumes expressed as a percentage. The implementation of a continuous MTA variable, compared to a five-step scale, might offer a more fine-grained metric to differentiate between abnormality and normality. Notwithstanding the presence of existing automated approaches for automated MTA scoring [20, 22, 31, 32, 34], successful integration is challenged by difficulties in accurately segmenting intricate anatomical structures (thus requiring manual correction) and accommodating to individual variations and unique patient characteristics [5, 7, 15, 35]. Therefore, continuous validation, evaluation, improvement, and complementation efforts are vital to address, augment, and overcome these complexities.

To this end, an ILV/hippocampus (Hip) ratio based on icobrain dm (dementia), a CE-marked and FDA-cleared automated volumetric post-processing software for clinical MRI scans, was developed [19, 36, 45]. The primary objective of this exploratory clinical study is to evaluate and compare the ILV/Hip ratio’s performance, to the hippocampal volumes and visual ratings, as part of the validation of automated volumetry in routine clinical practice. The secondary objective of this study is to investigate the correlation between visual MTA scales and the ILV/Hip ratio from icobrain dm in a heterogeneous patient population comprising different degrees of cognitive decline.

## Material and methods

### Study population

This study consisted of one-hundred-twelve subjects who underwent a clinical routine MRI examination in the context of a full cognitive clinical diagnostic work-up. All consented patients between the ages of 60 and 90 (inclusive) that had a memory consultation at the department of neurology at UZ Brussel between September 2020 and December 2022 were considered for inclusion, irrespective of the severity of cognitive decline. Exclusion criteria consisted of MRI contraindications and structural lesions in the region of interest (temporal lobe). Patient classification was effectuated in compliance with the National Institute on Aging-Alzheimer’s Association criteria for “MCI due to AD” and “Dementia due to AD” [1, 9, 17, 26, 44]. Subjective cognitive decline (SCD) subjects were diagnosed according to the criteria of Jessen’s et al. (2014) [21]. Note that the aforementioned criteria were applied wherever possible, since not all cerebrospinal fluid (CSF) biomarkers were available for the entire study population. Therefore, the final diagnosis used in this study does not necessarily imply a biomarker-based diagnosis. In total, 16 cognitively healthy controls (CN), 33 SCD subjects, 35 mild cognitive impairment patients (MCI), and 27 dementia (DEM) patients were included in this study. Lastly, a randomly selected patient with normal pressure hydrocephalus (NPH) was included to illustrate the effect of a large ILV volume on the MTA score.

### MRI acquisition protocol

Brain MRI was performed in all participants using the Philips Ingenia 3T (Philips Medical Systems, Best, The Netherlands). The MRI examination consisted of a sagittal 3D T1-weighted sequence, a sagittal 3D FLAIR-weighted sequence, a coronal T2-weighted sequence, a 3D susceptibility weighted imaging (SWI) and diffusion (DWI). For this study, only the 3D T1-weighted sequence for volumetry and MTA scoring on a coronal reconstruction was used. All scan parameters of the 3D T1-weighted sequence are listed in Supplementary Material Table 1.

### Image analysis

#### Visual assessment

The MTA scale by Scheltens, rated on coronal T1-weighted images, was determined individually by three experienced radiologists (G-J. A., T. V., and S. R.), blinded to diagnosis and sex. In case of discrepancy between individual ratings, a consensus MTA score was agreed upon. Images were viewed and evaluated on a Barco (Kortrijk, Belgium) diagnostic screen in AGFA Picture Archiving and Communication System (PACS).

#### Automated volumetry

From each T1-weighted image, automated brain volumetry was computed by icobrain dm (v 5.10) for total, left, and right hippocampal volumes, as well as for total, left, and right ILV volumes. The initial steps in icobrain dm’s pipeline included skull stripping, bias field correction, and computation of a head size normalization factor as the determinant of an affine transformation to MNI space. The hippocampal and ILV segmentations were obtained with a deep learning-based algorithm trained on a dataset of T1-weighted brain images with high variability both at the population level and in terms of scanners and acquisition parameters [28]. Additionally, a specific intensity-based augmentation strategy that enhances generalizability was used during training [27].

#### ILV/Hip ratio

An automated alternative of the visual MTA score showed the degree of hippocampal atrophy accounting for volume loss and compensatory expansion of the ILV, defined as the ratio between ILV and hippocampal volumes expressed as a percentage. The ILV/Hip ratio was calculated according to the following formula:

$$\frac{ILV}{Hip}ratio=\left(\frac{\left(Inferior\;lateral\;ventricle\;volume\left(\text{cm}^3\right),ILV\right)}{\left(Hippocampal\;volume\left(\text{cm}^3\right),HC\right)}\right)\times100$$

for each hemisphere (left and right) separately, as well as combined (total).

#### Normative reference population

In order to integrate the variables age and sex in the interpretation of the ILV/Hip ratio score, a large reference dataset (*n* = 1903, age range [min–max]: [6–96] years old) comprised subjects without cognitive complaints belonging to 14 different studies with participants derived from open-source data (Supplementary Material Table 2) was used to understand if an individuals’ ILV/Hip ratio score for each patient deviates from the expected score for an individual without cognitive complaints of the same age and sex. Normal aging, derived from the reference dataset, is modeled through univariate interpolating splines, fitted through the percentiles of a shifting age window. Comparing an ILV/Hip ratio score with the trends observed in the subjects without cognitive complaints resulted in a normative percentile adjusted for age and sex. This same methodology was also applied to hippocampal and ILV volumes, creating a percentile score that can be compared to a chosen “cut-off.” Typically, the range between percentile 1 and percentile 99 can be considered a normal range. Any value outside this range might be considered abnormal, which can be used in clinical routine to integrate age and thus evaluate whether a subject’s ILV/Hip ratio score deviates from a healthy aging pattern. A value between the 90 and 99th percentiles can still be considered normal, since this can be inherent to the normal distribution of biological variables, but should nevertheless be interpreted with caution, suggesting that clinical follow-up within 1–2 years might be warranted.

To demonstrate clinical interpretability on patient level, individual cases for each diagnostic category (CN, SCD, MCI, and DEM), as well as the NPH case, were presented. Lastly, an error bar (EB) calculation was performed to evaluate performance specifications, where the error bar interval (− EB and + EB) contains the difference between test and retest values with 90% confidence. This is important since automated measurements can be subject to measurement errors. Therefore, measurement variability should also be considered during result interpretation.

### Statistical analysis

#### Descriptive statistics

R environment (R-Studio, v.1.0.136) for statistical computing and graphics was used for all data processing with the following “packages” and (functions). Demographic information was reported as percentages, mean and standard deviation (SD) and/or median and interquartile range (*IQR*). For categorical variables, the chi-square test of independence was used, while continuous variables were analyzed by the ANOVA test, with a significance level of 0.05 (R package: “arsenal” (tableby and write2word)).

#### Inter-rater variability analysis

To ensure the quality of the visual assessment for an adequate comparison to automated volumetry, the inter-rater variability was evaluated through the intraclass correlation coefficient (*ICC*, 95% confidence intervals (*CI*)), a measure of reproducibility between repeated measurements of the same item, carried out by different observers. (R package “psych” (*ICC*, v. 2.3.0)). A two-way mixed model, single measurement, with absolute agreement measures was used. The output was the *ICC* estimate with its respective confidence intervals [38, 43]. The mean *ICC* and *CI* were calculated using Fisher’s *z* transformation (R function: (atanh), to transform the *ICC* values to *z*-scores, calculating the mean of the *z*-scores, and then applying the tanh() function to obtain the mean *ICC* value.

The *ICC* is a value going from 0, which indicates no agreement, to 1, indicating absolute agreement, which can be interpreted as either poor (*x* < 0.50), moderate (0.50 < *x* < 0.75), good (0.75 < *x* < 0.90), or excellent (*x* > 0.90), when taking into account the 95% confidence intervals of the *ICC* estimate, as suggested by Koo and Li in 2016 [2, 23]. The *ICC* was calculated using the following formula:

$$ICC=S2A/(S2A+S2W)$$

where *S*2*A* is the variance among groups, and *S*2*W* is the variance within groups [53]. The intra-rater variability was not assessed, as this was beyond the scope of this study.

#### Association analysis

The association between the visual MTA rating (each rater separately and the MTA consensus), cognitive outcome (reflected by the Mini-Mental State Examination, MMSE), and the automated brain segmentations computed by icobrain dm (total, left, and right hippocampal volumes, total, left, and right ILV volumes, and the total, left, and right ILV/Hip ratio) was first quantified using Spearman’s correlation analysis (R package: “base R” (cor)] (alpha < 0.05). Spearman’s rank correlation is a non-parametric measure with robustness to potential non-linearity and outliers, which is often encountered when comparing ordinal (visual MTA) and continuous (automated brain segmentation) variables. Spearman’s values range from − 1 to 1. A value of − 1 indicates a perfect negative monotonic relationship; 0 indicates no monotonic relationship; and 1 indicates a perfect positive monotonic relationship. The strength and direction of the relationship can be interpreted as follows: very weak (|*x*|< 0.20), weak (0.20 ≤|*x*|< 0.40), moderate (0.40 ≤|*x*|< 0.60), strong (0.60 ≤|*x*|< 0.80), or very strong (|*x*|≥ 0.80), where a positive value indicates parallel transitions, and a negative value implies an inverse relationship.

Additionally, for a more comprehensive analysis and to not be overly reliant on a single method, Kendall’s Tau (R package: “base R” (cor)] (alpha < 0.05) was used to give more weight to the ordinal nature of the visual MTA ratings and to examine the concordance between the automated measurements and visual MTA ratings. Kendall’s Tau, like Spearman’s correlation, is a versatile measure that ranges from − 1 to 1, where − 1 indicates a perfect negative association; 0 implies no association; and 1 signifies a perfect positive association. The strength and direction of the association can be summarized as very weak (|*x*|< 0.10), weak (0.10 ≤|*x*|< 0.30), moderate (0.30 ≤|*x*|< 0.50), strong (0.50 ≤|I|< 0.70), or very strong (|*x*|≥ 0.70).

Moreover, a logistic regression was performed (R package: “rms” (lrm)) to further evaluate the relationship between the visual MTA as outcome and the automated brain segmentations as predictor. Various additional scale invariant metrics, including the concordance index (c-index/area under the curve (*AUC*)) and the Brier score, were used to further quantitatively evaluate the strength, direction, degree of association, and correlation.

The AUC assesses how well the model distinguishes between the different visual MTA severity scores based on the predicted probabilities. The *AUC* varies being 0 and 1, whereas a general guideline *AUC* > 0.90 signifies excellent discrimination, 0.80 ≤ *AUC* < 0.90 represents good, 0.70 ≤ *AUC* < 0.80 denotes moderate to good, 0.60 ≤ *AUC* < 0.70 reflects moderate to poor, and *AUC* < 0.60 indicates very poor discrimination. *AUC* equals 0.50 indicates no discriminative power (random chance). Thus, a high *AUC* suggests the model is effective at ordering and ranking cases according to MTA severity and would indicate that both scoring methods provide very similar rankings, and by extension, high correlation, encouraging the prospects for interchangeability.

The Brier score quantifies the accuracy of predicted probabilities, where a low Brier score suggests accurate predictions, further reinforcing correlation between the two variables. A Brier score ≤ 0.2 indicates excellent model performance; 0.2 < Brier score ≤ 0.25 reflects a good; 0.25 < Brier score ≤ 0.3 suggests a fair; 0.3 < Brier score ≤ 0.35 signifies poor; and a Brier score > 0.35 implies very poor model performance.

Even though these additional metrics do not offer a traditional correlation coefficient, they do offer information about the quality of predictions and alignment between predicted and actual values, which indirectly reflects the degree of correlation.

#### Classification accuracy

To determine whether the ILV/Hip ratio exhibits comparable diagnostic precision or provides additional information compared to other measurements, classification accuracy through logistic regression of the visual MTA ratings (each rater separately and the MTA consensus) and the automated volumetric measurements (total, left, and right hippocampal volumes, total, left, and right ILV volumes, and the total, left, and right ILV/Hip ratio), was conducted for each variable separately as predictors. The following pairwise combinations of disease stages were considered as binary outcomes: SCD vs. CN, MCI vs. CN, DEM vs. CN, MCI vs. SCD, DEM vs. SCD, and DEM vs. MCI.

Classification performance was evaluated using receiver operating characteristic (ROC) analysis, with the R package “pROC” (roc, auc, coords, and ci) and the “stats” (predict and glm) package [40]. *AUC*, sensitivity, specificity, positive predictive value (*PPV*), and negative predictive value (*NPV*), were documented for each pairwise combination of disease stages. The *AUC* was computed with the trapezoidal rule. The Youden index to determine the threshold that maximizes the distance to the identity (diagonal) line, from which the sensitivity, specificity, *PPV*, and *NPV* were calculated. In addition, for each binary classification, resampling with replacement to estimate the variability of the *AUC* was employed. The resulting bootstrapped-based confidence intervals were then used to investigate if the *AUC* values between the variables were significantly different.

## Results

### Study population

The demographic and volumetric characteristics of the study population are presented in Table 1. This study population consisted of one-hundred-twelve subjects with a mean age (± *SD*) of 66.85 ± 13.64 years, composed of cognitively healthy controls (*N* = 16), SCD subjects (*N* = 33), and patients (MCI = 35, DEM = 27, and NPH = 1) belonging to different stages of cognitive decline.

**Table 1:** Demographic and volumetric characteristics of the study population

	CN (*N* = 16)	SCD (*N* = 33)	MCI (*N* = 35)	DEM (*N* = 27)	Total (*N* = 111)	*p*-value	*NPH (*N* = 1)
Sex — %F	64.3	57.6	37.1	63.0	53.2	0.133	0
Age (years)							
Mean (*SD*)	58.1 (16.6)	58.6 (11.6)	72.6 (12.6)	70.1 (6.4)	66.3 (12.90)	< .001	73.7 (NA)
Median [*IQR*]	60.5 [47.1,72.6]	58.8 [50.6,64.8]	76.3 [70.1,81.8]	73.1 [67.5,76.1]	68.7[58.79, 76.45]		73.7 [73.7,73.7]
MMSE (0–30)							
#	2	9	25	25	61		
Mean (*SD*)	29 (0)	29 (1)	26 (2)	20 (6)	24 (5)	< .011	NA (NA)
Median [*IQR*]	29 [29, 29]	29 [28, 29]	27 [26, 28]	20 [19, 23]	26 [22, 28]		NA [NA, NA]
Hip (mL) — total							
Mean (*SD*)	9.086 (0.659)	9.118 (0.729)	8.172 (1.017)	7.513 (1.163)	8.413 (1.137)	< .001	7.391 (NA)
Median [*IQR*]	9.024 [8.772,9.672]	9.241 [8.564, 9.601]	8.318 [7.734, 8.818]	7.463 [6.825, 8.389]	8.522 [7.881, 9.303]		7.391 [7.391, 7.391]
Hip (mL) — left							
Mean (*SD*)	4.449 (0.343)	4.442 (0.382)	3.981 (0.474)	3.668 (0.646)	4.103 (0.575)	< .001	3.362 (NA)
Median [*IQR*]	4.369 [4.270, 4.613]	4.514 [4.144, 4.655]	3.995 [3.811, 4.278]	3.733 [3.275, 4.126]	4.176 [3.861, 4.522]		3.362 [3.362, 3.362]
Hip (mL) — right							
Mean (*SD*)	4.637 (0.350)	4.676 (0.379)	4.192 (0.573)	3.845 (0.586)	4.310 (0.597)	< .001	4.030 (NA)
Median [*IQR*]	4.672 [4.498, 4.879]	4.742 [4.451, 4.960]	4.280 [3.974, 4.544]	3.841 [3.488, 4.155]	4.390 [4.003, 4.779]		4.030 [4.030, 4.030]
ILV (mL) — total							
Mean (*SD*)	2.293 (1.605)	1.984 (1.538)	3.540 (1.918)	5.228 (1.717)	3.327 (2.118)	< .001	7.949 (NA)
Median [*IQR*]	1.982 [1.360, 2.430]	1.378 [1.024, 2.522]	3.094 [2.007, 4.534]	4.687 [4.018, 5.921]	2.936 [1.430, 4.637]		9.674 [9.674, 9.674]
ILV (mL) — left							
Mean (*SD*)	1.200 (0.749)	1.137 (0.789)	1.917 (1.035)	2.680 (0.887)	1.778 (1.076)	< .001	4.950 (NA)
Median [*IQR*]	1.152 [0.649, 1.433]	0.829 [0.658, 1.644]	1.627 [1.167, 2.417]	2.447 [2.158, 3.223]	1.463 [0.873, 2.457]		4.950 [4.950, 4.950]
ILV (mL) — right							
Mean (*SD*)	1.070 (0.878)	0.824 (0.781)	1.577 (0.906)	2.485 (1.033)	1.509 (1.094)	< .001	4.721 (NA)
Median [*IQR*]	0.846 [0.548, 1.142]	0.498 [0.381, 0.973]	1.526 [0.876, 2.027]	2.329 [1.592, 3.132]	1.238 [0.562, 2.103]		4.721 [4.721, 4.721]
ILV/Hip ratio (%) — total							
Mean (*SD*)	25.880 (18.936)	22.419(18.537)	46.251 (31.049)	73.109 (31.893)	43.072 (33.028)	< .001	130.877 (NA)
Median [*IQR*]	22.434 [14.510, 27.737]	15.068 [10.484,28.808]	38.500 [23.847,56.501]	67.034 [51.694,85.674]	33.236 [16.268, 60.570]		130.877 [130.877, 130.877]
ILV/Hip ratio (%) — left							
Mean (*SD*)	27.518 (17.777)	26.211 (19.182)	50.653 (31.934)	77.521 (34.528)	46.937 (34.257)	< .001	146.247 (NA)
Median [*IQR*]	26.811 [13.435, 32.478]	18.723 [14.536, 36.416]	38.533 [26.907, 63.184]	68.683 [56.887, 101.155]	37.792 [19.576, 65.260]		146.247 [146.247, 146.247]
ILV/Hip ratio (%) — right							
Mean (*SD*)	23.863 (20.563)	18.405 (19.117)	41.112 (31.326)	68.768 (38.215)	38.873 (34.669)	< .001	117.172 (NA)
Median [*IQR*]	18.676 [11.287, 25.527]	10.605 [7.968, 19.180]	37.634 [20.195, 48.829]	62.437 [40.802, 80.161]	28.270 [12.693, 49.645]		117.172 [117.172, 117.172]
Visual MTA — total							
Mean (*SD*)	0 (0)	0 (1)	1 (1)	2 (1)	1 (1)	< .001	4 (NA)
Median [*IQR*]	0 [0,1]	0 [0,1]	1 [1,2]	2 [1,3]	1 [0,2]		4 [4,4]
Visual MTA — left							
Mean (*SD*)	0 (0)	0 (0)	1 (1)	2 (1)	1 (1)	< .001	4 (NA)
Median [*IQR*]	0 [0,0]	0 [0,1]	1 [1,2]	2 [2,3]	1 [0,2]		4 [4,4]
Visual MTA — right							
Mean (*SD*)	0 (0)	0 (1)	1 (1)	2 (1)	1 (1)	< .001	4 (NA)
Median [*IQR*]	0 [0,0]	0 [0,1]	1 [1,2]	2 [2,3]	1 [0,2]		4 [4,4]

Data description as mean and standard deviation (*SD*), median [interquartile range, *IQR*] and/or percentages, where applicable. Chi-Square test (categorical variables), ANOVA analysis (continuous variables). Brain volumes are normalized for head size. *BL* baseline. *CN* cognitively healthy controls, *SCD* subjective cognitive decline subjects, *MCI* mild cognitive impairment patients, *DEM* dementia, *NPH* normal pressure hydrocephalus, *MTA* medial temporal lobe atrophy, *ILV* Inferior lateral ventricle, *Hip* hippocampal volume. *MMSE* Mini-Mental State Examination

^*^ Since the NPH patient was included in this study to show the additional validity of the ILV/Hip ratio score, it was excluded from the statistical analysis of the core study population

### Inter-rater variability of visual assessment

To assess visual assessment reproducibility, the inter-rater variability for each visually rated MTA score (total, left, and right) was determined for all pairwise and all-rater comparisons (Table 2). The largest inter-rater variability was found between Rater I and Rater II for the right MTA score (0.714 [0.610, 0.794]). The smallest overall inter-rater variability was seen between Rater I and Rater III for the total MTA score (0.907 [0.868, 0.935]).

**Table 2:** Inter-rater variability

	Rater I vs. Rater II	Rater II vs. Rater III	Rater I vs. Rater III	All rater comparison
MTA — total	0.790 [0.709, 0.850]	0.820 [0.749, 0.873]	0.907 [0.868, 0.935]	0.840 [0.789, 0.882]
MTA — left	0.788 [0.706, 0.849]	0.807 [0.732, 0.863]	0.861 [0.805, 0.903]	0.819 [0.763, 0.866]
MTA — right	0.714 [0.610, 0.794]	0.785 [0.702, 0.847]	0.856 [0.799, 0.899]	0.785 [0.732, 0.840]
MEAN ICC	0.766 [0.715, 0.809]	0.804 [0.784, 0.823]	0.877 [0.838, 0.907]	0.816 [0.782, 0.845]

Inter-rater variability was calculated using the intraclass correlation coefficient (*ICC*, [95% confidence interval, *CI*]) for the following rater combinations: Rater I vs. Rater II, Rater II vs. Rater III, Rater I vs. Rater III, as well as an all-rater comparison, for total, left, and right visual MTA scores

***MTA*** medial temporal lobe atrophy

### Relationship between automated volumetry and the visual MTA score

**Fig. 1:**
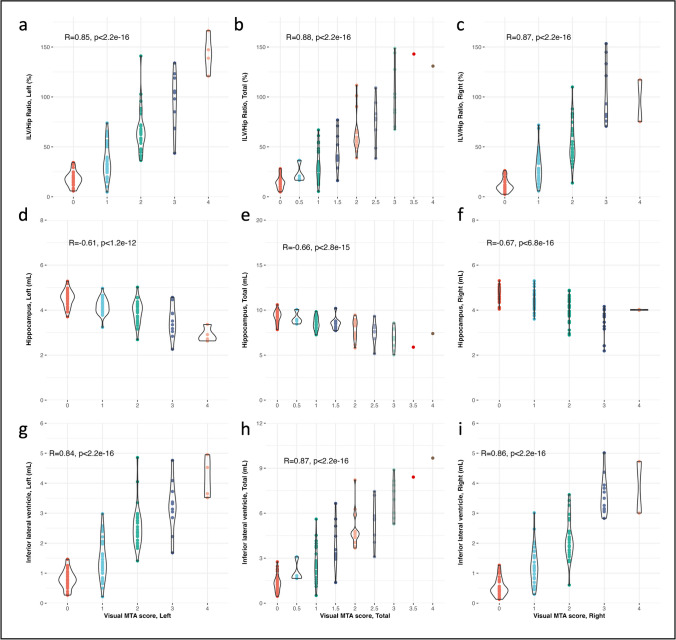
Violin plots of visually assessed medial temporal atrophy (MTA) score and ILV/Hip ratio (left: **a**, total: **b**, and right: **c**), hippocampal volumes (left: **d**, total: **e**, and right: **f**), and inferior lateral ventricular (ILV) volumes (left: **g**, total: **h**, and right: **i**)

**Fig. 2:**
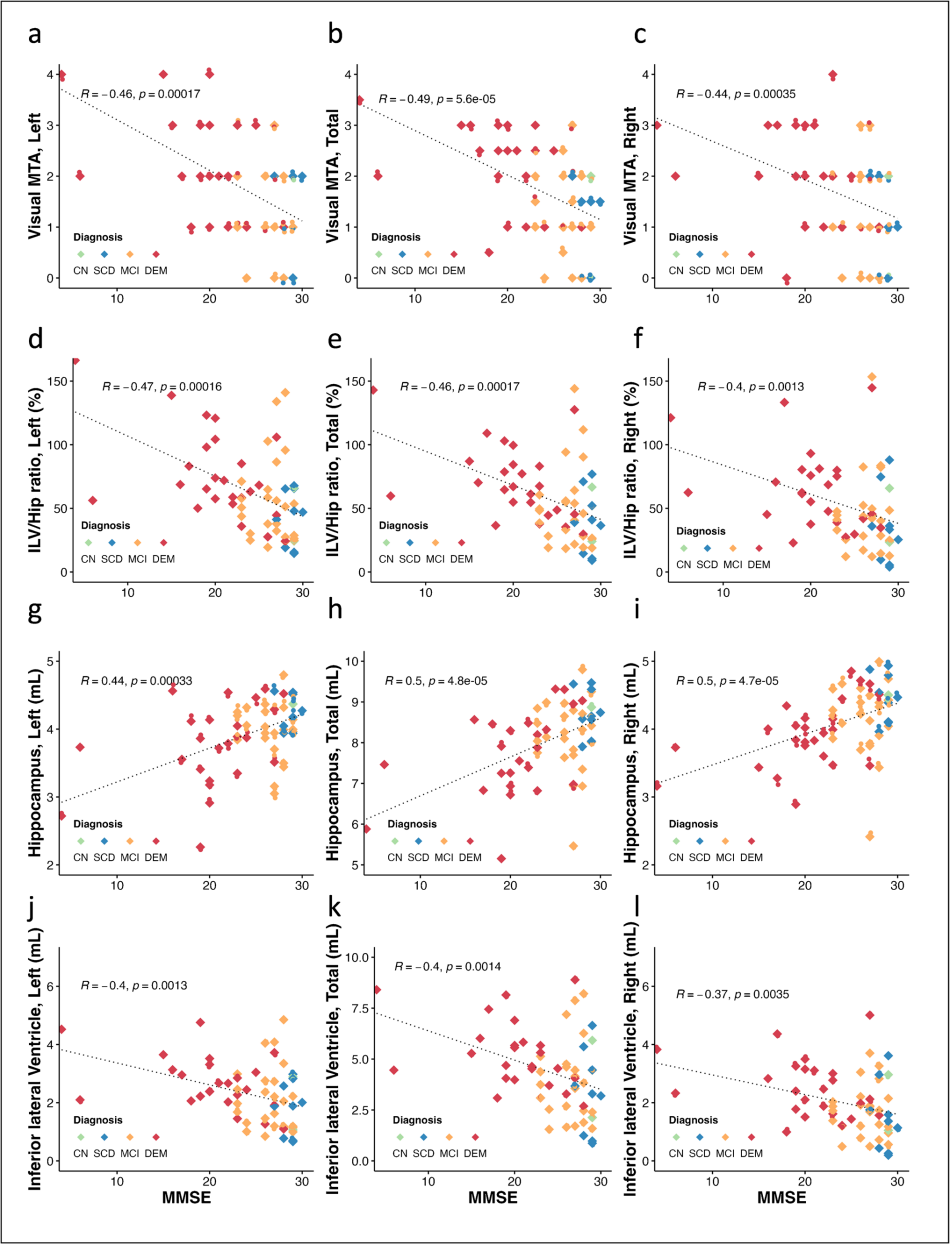
Spearman’s correlations graphs of cognitive outcome reflected by the Mini-Mental State Examination (MMSE) versus visually assessed medial temporal atrophy (MTA) scores (left: **a**, total: **b**, and right: **c**), ILV/Hip ratio (left: **d**, total: **e**, and right: **f**), hippocampal volumes (left: g, total: h, and right: i), and inferior lateral ventricular (ILV) volumes (left: **j**, total: **k**, and right: **l**)

Subsequently, the association between visual MTA scores (each rater separately and the MTA consensus), cognitive outcome (reflected by the MMSE), and the automated brain segmentations computed by icobrain dm (total, left, and right hippocampal volumes, total, left, and right ILV volumes, and total, left, and right ILV/Hip ratio) were assessed using ordinal logistic regression, the Brier’s score, Kendall Tau, and Spearman’s correlation analysis (Table 3). Additionally, the automated brain structure volumes and calculated ILV/Hip ratio versus the consensus MTA rating scores were visualized per MTA score (0–4) (Fig. 1). The automated measurements and consensus MTA scores versus the MMSE were visualized in Fig. 2.

**Table 3:** The relationship between visual MTA and automated volumetry

Comparison	Rater	*Coeff*	*S. E*	Wald-Z	*AUC*	Brier score	Kendall-Tau	Spearman rho
Visual MTA, left vs								
Hip, left (mL)	Rater I	− 2.519	0.391	− 6,44	0.758	0.162	− 0.440	− 0.554
	Rater II	− 3.317	0.455	− 7.30	0.805	0.175	− 0.526	− 0.657
	Rater III	− 2.412	0.387	− 6.24	0.745	0.182	− 0.423	− 0.536
	Consensus	− 2.957	0.426	− 6.95	0.784	0.168	− 0.488	− 0.608
ILV, left (mL)	Rater I	2.461	0.315	7.81	0.875	0.102	0.638	0.780
	Rater II	2.198	0.279	7.87	0.868	0.099	0.635	0.785
	Rater III	2.241	0.279	8.02	0.872	0.103	0.642	0.780
	Consensus	2.729	0.337	8.09	0.904	0.088	0.695	0.836
ILV/Hip, left (%)	Rater I	0.082	0.011	7.75	0.884	0.099	0.654	0.793
	Rater II	0.085	0.011	7.97	0.887	0.097	0.668	0.811
	Rater III	0.076	0.001	7.97	0.878	0.107	0.652	0.787
	**Consensus**	**0.100**	**0.013**	**7.95**	**0.915**	**0.088**	**0.715**	**0.851**
Visual MTA, right vs								
Hip, right (mL)	Rater I	− 2.327	0.368	− 6.33	0.786	0.154	− 0.486	− 0.617
	Rater II	− 2.735	0.394	− 6.94	0.825	0.149	− 0.564	− 0.712
	Rater III	− 2.171	0.348	− 6.23	0.776	0.164	− 0.477	− 0.611
	Consensus	− 2.560	0.377	− 6.78	0.807	0.152	− 0.526	− 0.670
ILV, right (mL)	Rater I	2.346	0.301	7.80	0.880	0.120	0.645	0.781
	Rater II	1.624	0.225	7.22	0.839	0.123	0.587	0.746
	Rater III	2.544	0.313	8.12	0.911	0.090	0.708	0.852
	Consensus	3.227	0.413	7.81	0.928	0.087	0.733	0.864
ILV/Hip, right (%)	Rater I	0.068	0.009	7.20	0.883	0.114	0.650	0.789
	Rater II	0.048	0.007	6.62	0.854	0.128	0.614	0.771
	Rater III	0.069	0.009	7.47	0.908	0.097	0.703	0.850
	**Consensus**	**0.095**	**0.013**	**7./19**	**0.929**	**0.089**	**0.734**	**0.866**
Visual MTA, total vs								
Hip, total (mL)	Rater I	− 1.256	0.186	− 6.75	0.761	0.137	− 0.463	− 0.602
	Rater II	− 1.636	0.210	− 7.79	0.796	0.138	− 0.549	− 0.716
	Rater III	− 1.182	0.815	− 6.51	0.743	0.166	− 0.440	− 0.581
	Consensus	− 1.459	0.201	− 7.27	0.782	0.139	− 0.508	− 0.659
ILV, total (mL)	Rater I	1.261	0.147	8.57	0.868	0.091	0.654	0.801
	Rater II	0.971	0.117	8.28	0.828	0.109	0.608	0.783
	Rater III	1.243	0.139	8.93	0.878	0.085	0.685	0.840
	Consensus	1.528	0.171	8.96	0.900	0.083	0.721	0.867
ILV/Hip, total (%)	Rater I	0.080	0.010	8.35	0.874	0.085	0.663	0.813
	Rater II	0.065	0.008	8.18	0.845	0.102	0.639	0.810
	Rater III	0.077	0.009	8.49	0.880	0.092	0.690	0.843
	**Consensus**	**0.102**	**0.012**	**8.55**	**0.906**	**0.082**	**0.733**	**0.877**

The relationship between different automated methods (only Hip (hippocampal volume), only ILV (inferior lateral ventricle), and ILV/Hip ratio) was compared against the medial temporal lobe atrophy (MTA) consensus and individual raters’ scores. Total volumes, as well as left and right hemispheres, were analyzed. All Spearman and Kendall-Tau correlations were statistically significant (*p *< .001). The automated method displaying the strongest association to each (total, left, or right) visual MTA rating was highlighted in bold

*S. E.* standard error, *AUC* area under the curve

A high consistency was observed across the various association metrics testing for potential interchangeability among the considered variables. Within both individual raters’ analyses and the consensus, the consensus scores demonstrated the strongest correlation to the automated measurements. Specifically, the total, left, and right ILV/Hip ratio within the consensus scores displayed the most robust associations with the corresponding total, left, and right visual MTA ratings. Moreover, the ILV/Hip ratio showed a uniform pattern of higher AUC, Kendall-Tau, and Spearman coefficients, along with lower Brier scores, in comparison to the correlation between visual MTA ratings and the ILV, or hippocampal volumes alone.

The Spearman’s correlation analysis indicated a moderate to strong negative significant (*p* < 0.001) correlation for total (*ρ* =  − 0.659), left (*ρ* =  − 0.608), and right (*ρ* =  − 0.670) hippocampal volumes versus the consensus MTA ratings. Conversely, a strong positive and significant (*p* < 0.001) correlation was seen for the total (*ρ* = 0.867), left (*ρ* = 0.836), and right (*ρ* = 0.864) ILV volumes versus the consensus MTA rating, comparable to the total (*ρ* = 0.877), left (*ρ* = 0.851), and right (*ρ* = 0.866) ILV/Hip ratio score versus the consensus MTA rating. It is noteworthy that, while individual hippocampal volumes showed the lowest Spearman’s correlation to the MTA score, individual ILV volumes exhibited a slightly lower correlation to the MMSE score (total (*ρ* =  − 0.400), left (*ρ* =  − 0.403), and right (*ρ* =  − 0.368) compared to the other examined measures (visual MTA: total (*ρ* =  − 0.492), left (*ρ* =  − 0.463), and right (*ρ* =  − 0.443), ILV/Hip ratio: total (*ρ* =  − 0.464), left (*ρ* =  − 0.466), and right (*ρ* =  − 0.402), and hippocampus: total (*ρ* = 0.496), left (*ρ* = 0.445), and right (*ρ* = 0.497)), respectively.

The Kendall Tau analysis provided an additional complementary layer of confirmation, consistently mirroring the patterns previously observed in Spearman’s correlation. The concordance among different correlation measures suggests the independence of observed relationships from specific data characteristics and underscores the reliability of the interconnectedness between variables, regardless of the analytical approach chosen.

### Classification accuracy

The classification accuracy of for the automated brain segmentations and the visual MTA consensus ratings are listed in Table 4. The ILV/hip ratio measurements demonstrate competitive classification performance to the visual MTA scores and varying levels of sensitivity and specificity across different pairwise comparisons. No significant differences were observed between the two methods based on to the confidence intervals of the *AUC* values. As the disease severity gap widens from milder stages (e.g., SCD vs. CN) to more severe stages (e.g., DEM vs. CN)), there is a notable trend of increasing *AUC*, improved sensitivity, and specificity.

**Table 4:** Classification accuracy

Method	Pairwise comparison	Sensitivity	Specificity	*AUC* [95% *CI*]	*PPV*	*NPV*
Visual MTA						
MTA, left	SCD vs. CN	0.485	0.686	0.599 [0.455–0.744]	0.608	0.572
	MCI vs. CN	0.829	0.688	0.801 [0.680–0.922]	0.726	0.800
	**DEM vs. CN**	**0.999**	**0.688**	**0.941 [0.878–0.999]**	**0.762**	**0.999**
	MCI vs. SCD	0.829	0.515	0.706 [0.588–0.823]	0.631	0.750
	DEM vs. SCD	0.741	0.818	0.883 [0.809–0.958]	0.803	0.759
	DEM vs. MCI	0.407	0.943	0.748 [0.632–0.863]	0.877	0.614
MTA, right	SCD vs. CN	0.515	0.750	0.635 [0.494–0.777]	0.673	0.607
	MCI vs. CN	0.800	0.750	0.802 [0.681–0.923]	0.762	0.789
	**DEM vs. CN**	**0.778**	**0.938**	**0.935 [0.865–0.999]**	**0.927**	**0.808**
	MCI vs. SCD	0.801	0.485	0.689 [0.571–0.808]	0.608	0.708
	DEM vs. SCD	0.778	0.848	0.879 [0.796–0.961]	0.837	0.792
	DEM vs. MCI	0.778	0.600	0.753 [0.637–0.869]	0.660	0.730
MTA, total	SCD vs. CN	0.484	0.750	0.625 [0.480–0.770]	0.659	0.592
	MCI vs. CN	0.800	0.750	0.812 [0.691–0.932]	0.762	0.789
	**DEM vs. CN**	**0.778**	**0.938**	**0.953 [0.898–0.999]**	**0.926**	**0.808**
	MCI vs. SCD	0.800	0.515	0.703 [0.582–0.823]	0.623	0.720
	DEM vs. SCD	0.741	0.939	0.900 [0.825–0.974	0.924	0.784
	DEM vs. MCI	0.556	0.914	0.779 [0.663–0.895]	0.866	0.673
ILV						
ILV, left (mL)	SCD vs. CN	0.606	0.625	0.538 [0.362–0.714]	0.618	0.614
	MCI vs. CN	0.543	0.875	0.734 [0.581–0.887]	0.813	0.657
	DEM vs. CN	0.889	0.875	0.905 [0.803–0.999]	0.877	0.887
	MCI vs. SCD	0.801	0.697	0.758 [0.639–0.876]	0.725	0.777
	**DEM vs. SCD**	**0.889**	**0.849**	**0.908 [0.833–0.983]**	**0.854**	**0.884**
	DEM vs. MCI	0.852	0.601	0.735 [0.610–0.861]	0.680	0.802
ILV, right (mL)	SCD vs. CN	0.697	0.563	0.585 [0.408–0.762]	0.614	0.650
	MCI vs. CN	0.686	0.813	0.723 [0.565–0.882]	0.785	0.720
	DEM vs. CN	0.926	0.875	0.903 [0.786–0.999]	0.881	0.922
	MCI vs. SCD	0.686	0.818	0.781 [0.669–0.893]	0.790	0.722
	**DEM vs. SCD**	**0.963**	**0.818**	**0.921 [0.849–0.993]**	**0.841**	**0.957**
	DEM vs. MCI	0.926	0.486	0.746 [0.625–0.867]	0.643	0.868
ILV, total (mL)	SCD vs. CN	0.394	0.813	0.576 [0.406–0.745]	0.678	0.573
	MCI vs. CN	0.686	0.813	0.739 [0.583–0.895]	0.785	0.720
	DEM vs. CN	0.963	0.850	0.912 [0.795–0.999]	0.885	0.959
	MCI vs. SCD	0.743	0.727	0.766 [0.651–0.882]	0.731	0.739
	**DEM vs. SCD**	**0.999**	**0.758**	**0.917 [0.845–0.990]**	**0.805**	**0.999**
	DEM vs. MCI	0.963	0.486	0.757 [0.638–0.876]	0.651	0.929
Hippocampus						
Hip, left (mL)	SCD vs. CN	0.333	0.813	0.496 [0.325–0.668]	0.640	0.549
	MCI vs. CN	0.600	0.938	0.793 [0.664–0.921]	0.906	0.701
	**DEM vs. CN**	**0.740**	**0.938**	**0.850 [0.736–0.964]**	**0.922**	**0.783**
	MCI vs. SCD	0.829	0.606	0.762 [0.649–0.875]	0.678	0.780
	DEM vs. SCD	0.667	0.970	0.846 [0.746–0.947]	0.957	0.744
	DEM vs. MCI	0.667	0.686	0.644 [0.498–0.791]	0.680	0.673
Hip, right (mL)	SCD vs. CN	0.333	0.875	0.546 [0.377–0.714]	0.727	0.568
	MCI vs. CN	0.743	0.750	0.750 [0.609–0.892]	0.748	0.745
	DEM vs. CN	0.778	0.936	0.887 [0.789–0.984]	0.926	0.808
	MCI vs. SCD	0.714	0.758	0.764 [0.650–0.877]	0.747	0.726
	**DEM vs. SCD**	**0.704**	**0.939**	**0.897 [0.819–0.975]**	**0.921**	**0.760**
	DEM vs. MCI	0.704	0.714	0.691 [0.554–0.828]	0.711	0.707
Hip, total (mL)	SCD vs. CN	0.151	0.999	0.510 [0.343–0.683]	0.999	0.541
	MCI vs. CN	0.543	0.938	0.783 [0.651–0.917]	0.897	0.672
	DEM vs. CN	0.741	0.938	0.882 [0.784–0.980]	0.922	0.783
	MCI vs. SCD	0.601	0.819	0.771 [0.660–0.881]	0.767	0.672
	**DEM vs. SCD**	**0.741**	**0.879**	**0.887 [0.805–0.968]**	**0.859**	**0.772**
	DEM vs. MCI	0.556	0.829	0.672 [0.533–0.811]	0.764	0.651
ILV/Hip ratio						
ILV/Hip, left (%)	SCD vs. CN	0.484	0.688	0.534 [0.360–0.708]	0.608	0.572
	MCI vs. CN	0.601	0.875	0.759 [0.615–0.903]	0.828	0.686
	**DEM vs. CN**	**0.999**	**0.688**	**0.941 [0.878–0.999]**	**0.762**	**0.999**
	MCI vs. SCD	0.857	0.667	0.773 [0.658–0.887]	0.720	0.824
	DEM vs. SCD	0.852	0.879	0.920 [0.855–0.986]	0.875	0.855
	DEM vs. MCI	0.778	0.657	0.745 [0.621–0.869]	0.694	0.747
ILV/Hip, right (%)	SCD vs. CN	0.697	0.563	0.593 [0.418–0.768]	0.614	0.650
	MCI vs. CN	0.714	0.750	0.739 [0.586–0.893]	0.740	0.724
	DEM vs. CN	0.778	0.938	0.935 [0.865–0.999]	0.926	0.808
	MCI vs. SCD	0.743	0.788	0.789 [0.680–0.899]	0.778	0.754
	**DEM vs. SCD**	**0.999**	**0.787**	**0.935 [0.895–0.999]**	**0.825**	**0.999**
	DEM vs. MCI	0.593	0.829	0.739 [0.615–0.863]	0.776	0.670
ILV/Hip, total (%)	SCD vs. CN	0.303	0.875	0.563 [0.391–0.734]	0.708	0.557
	MCI vs. CN	0.686	0.813	0.746 [0.593–0.900]	0.785	0.721
	**DEM vs. CN**	**0.963**	**0.875**	**0.938 [0.853–0.999]**	**0.885**	**0.959**
	MCI vs. SCD	0.886	0.667	0.785 [0.674–0.896]	0.726	0.854
	DEM vs. SCD	0.999	0,788	0.933 [0.872–0.994]	0.825	0.999
	DEM vs. MCI	0.741	0.686	0.760 [0.640–0.879]	0.702	0.726

The classification accuracy, presented as sensitivity and specificity at the Youden index, *AUC* with bootstrapped *CI*, *PPV*, and *NPV* at the Youden index, of three different automated methods (only Hip (hippocampal volume), only ILV (inferior lateral ventricle), and ILV/Hip ratio)), and the medial temporal lobe atrophy (MTA) consensus rating for different pairwise comparisons was reported. Total volumes, as well as left and right hemispheres, were analyzed. For each automated method, the pairwise comparisons showing the highest AUCs were highlighted in bold

*CN* cognitively normal control, *SCD* subjective cognitive decline subjects, *MCI* mild cognitive impairment patients, *DEM* dementia patients, *AUC* area under the curve, *CI* confidence interval

Particularly, when assessing DEM vs. CN, both the visual MTA and total ILV/Hip ratio showed an excellent comparable performance of (*AUC* [*CI*]) 0.953 [0.898–0.999] with a sensitivity of 0.778, and a specificity of 0.938 for the total visual MTA consensus rating, and a corresponding *AUC* of 0.938 [0.853–0.999], sensitivity of 0.963, and specificity of 0.875 for the total ILV/Hip ratio, respectively. Similarly, the individual ILV volumes demonstrated excellent discriminative power, with an *AUC* of 0.912 [0.795–0.999], sensitivity of 0.963, and specificity of 0.850. The individual hippocampal volumes showcased a slightly lower, but still strong performance, with an *AUC* of 0.882 [0.784–0.980], sensitivity of 0.741, and a specificity of 0.938. Interestingly, individual total ILV volumes exhibited a high specificity of 0.960, but a notably lower specificity of 0.486 for the DEM vs. MCI pairwise comparison, compared to a well-balanced sensitivity of 0.740 and specificity of 0.686 for the total ILV/Hip ratio. Moreover, total hippocampal volumes showed a comparable pattern in specificity, but a consistently lower sensitivity compared to the total ILV/Hip ratio. Recall that all sensitivity and specificity values were computed at the Youden index, which optimizes the sum between sensitivity and specificity; other trade-offs between sensitivity and specificity would be obtained by using different cut-off selection methods.

The ILV/Hip ratio and individual ILV volumes performing similarly, with slightly lower outcomes observed for hippocampal volumes, is a consistent trend across all pairwise comparisons, emphasizing a commensurable performance in terms of classification accuracy for automated brain segmentations and visual MTA consensus ratings.

### Comparison to a normative reference population

**Fig. 3:**
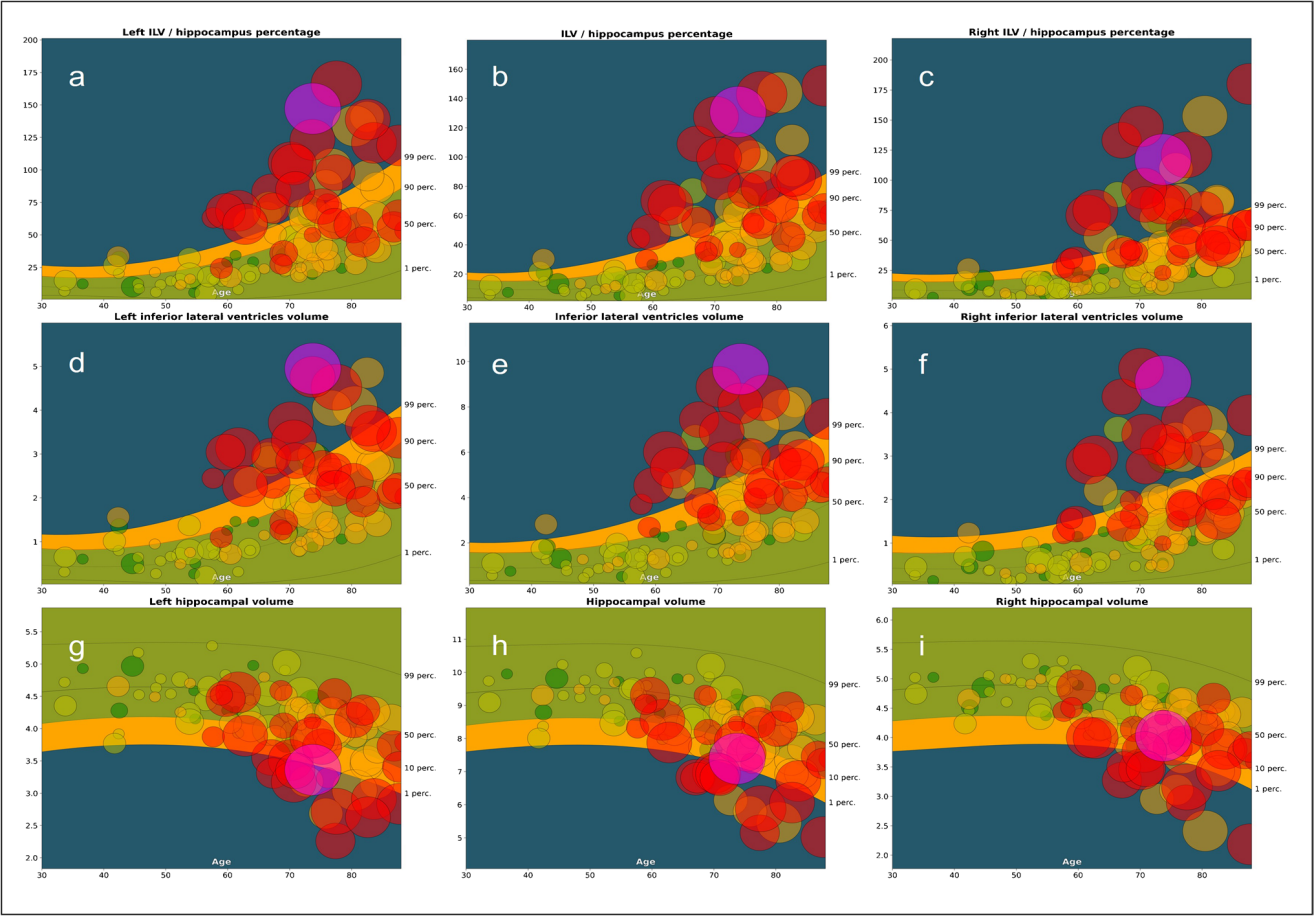
Population graphs — ILV/Hip ratio (in %, left: **a**, total: **b**, and right: **c**), inferior lateral ventricular (ILV) volumes (in mL, left: **d,** total: **e**, and right: **f)** and hippocampal volumes (in mL, left: **g**, total: **h**, and right: **i**). The graphs show the median and normal ranges (percentiles 1 to 99, with percentiles 90, 50, and 10 also represented) of selected brain structure volumes/ratios of cognitively healthy subjects (*n* = 1903) across a relevant age interval. The time point volumes are marked as circles. The size of the circles corresponds to the Scheltens’ MTA consensus score given by the three radiologists, which MTA = 0 corresponding to the smallest circle size, and MTA = 4 to the largest circle size. Color-coded dot representation; CN, dark green; SCD, green; MCI, orange, DEM, red; and NPH, purple). Color-coded population graph percentile interpretation; 1–90th percentile, green (normal); 90–99th percentile, orange (caution); and > 99th percentile, dark blue (abnormality)

Subsequently, all automated segmentations were compared to a reference population of subjects without cognitive complaints (*n* = 1903), depicting the normal ranges of selected brain structure volumes/ratios across a relevant age interval (Fig. 3). Performance specifications, featuring error bar results, can be found in Supplementary Material Table 3.

For every individual, the ILV/hip ratio was juxtaposed with the normative reference population. Individual values are illustrated by dots on the graph and the reference population is represented through a color-coded background and percentile lines indicated on the right side of the graph. Each individual’s dot size corresponded to their visual MTA consensus score, with larger circles denoting higher MTA scores. When looking at the ILV/Hip ratio population graphs, a clear correlation with increasing MTA scores (dot size), diagnosis (color-coded), and reference population percentiles is visible. The classification of MTA “severity” was proportional between visual MTA ratings and the percentiles of ILV/Hip ratio measurements on the population graphs, indicating an equipollent performance of visual assessment and automated volumetry for MTA determination. When looking at the NPH case, it is more likely that the MTA severity is caused by a deviation of ILV volumes (Fig. 2d–f, purple circle located in the blue zone) and not by an abnormal hippocampal volume (Fig. 2g–i, purple circle located in the orange zone). Individual percentiles per subject can be found in Supplementary Material Table 4.

### Clinical interpretability at patient level

To validate clinical interpretability at patient level, a subject was selected from each diagnostic category and assessed individually. The following brain structures were evaluated: upper lateral ventricles (green), inferior lateral ventricles (purple), and hippocampal volumes (yellow).

**Fig. 4:**
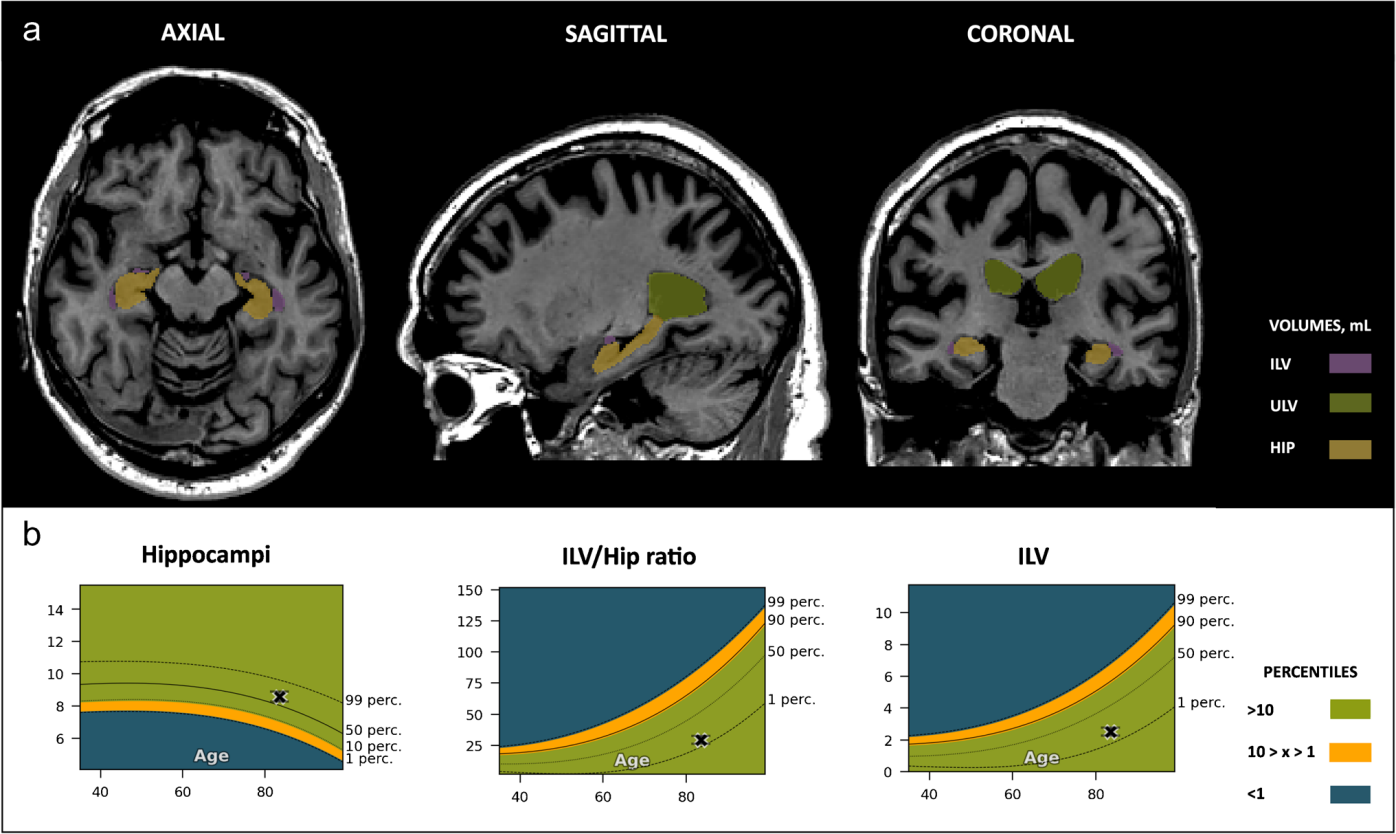
Cognitively normal (CN) case. Cross-sectional coronal T1-weighted image at the level of the medial temporal lobe and hippocampus with segmentation of the upper lateral ventricles (green), inferior lateral ventricle (purple) and hippocampus (yellow). **A** — Automated volumetric measurements *left*: Axial view *middle*: Sagittal view *right*: Coronal view. **B** — Population graphs *left*: hippocampal volumes of this subject showing the gray “x” marker in the green region just above between the 50th and 90th percentiles; *middle*: the ILV/Hip ratio of this subject showing the gray “x” marker in the green region between the 1st and 50th percentiles. *Right*: ILV volumes of this subject showing the gray “x” marker in the green region between the 1st and 50th percentiles. Color-coded population graph percentile interpretation; 1–90th percentiles, green (normal); 90–99th percentiles, orange (caution); and > 99th percentile, dark blue (abnormality)

In the case of the CN (age, 83.3 years old) subject, all automated measurements fell within the 1st to the 90th percentile range when compared to an age-matched reference population of healthy individuals (Fig. 4). This aligns with the visual assessment, which yielded an MTA score of 1 for both left and right hemispheres, as well as the total MTA score.

**Fig. 5:**
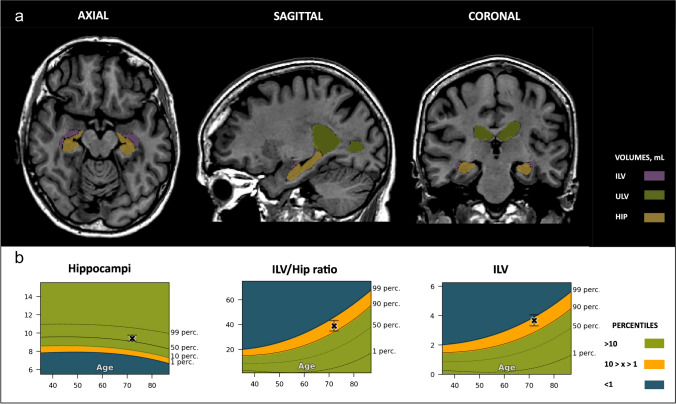
Subjective cognitive decline (SCD) case. Cross-sectional coronal T1-weighted image at the level of the medial temporal lobe and hippocampus with segmentation of the upper lateral ventricles (green), inferior lateral ventricle (purple), and hippocampus (yellow). **A** — Automated volumetric measurements *left*: axial view; *middle*: sagittal view; and *right*: coronal view. **B** — Population graphs *left*: hippocampal volumes of this subject showing the gray “x” marker in the green region just above the 50th percentile; *middle*: the ILV/Hip ratio of this subject showing the gray “x” marker in the orange region between the 90th and 99th percentiles. *Right*: ILV volumes of this subject showing the gray “x” marker in the orange region between the 90th and 99th percentiles. Color-coded population graph percentile interpretation; 1–90th percentile, green (normal); 90–99th percentile, orange (caution); > 99th percentile, dark blue (abnormality)

For the SCD subject (age, 71.9 years old), the hippocampal brain structure segmentations demonstrated values between the 50th and 90th percentile range, which is within the expected normal range compared to a population of similar age. In contrast, the ILV and ILV/Hip ratio fell between the 90–99th percentile, suggesting caution. Visual inspection yielded a consistent rating of 2 across all MTA measurements, which, taking the subject’s age into account, was considered abnormal (Fig. 5).

**Fig. 6:**
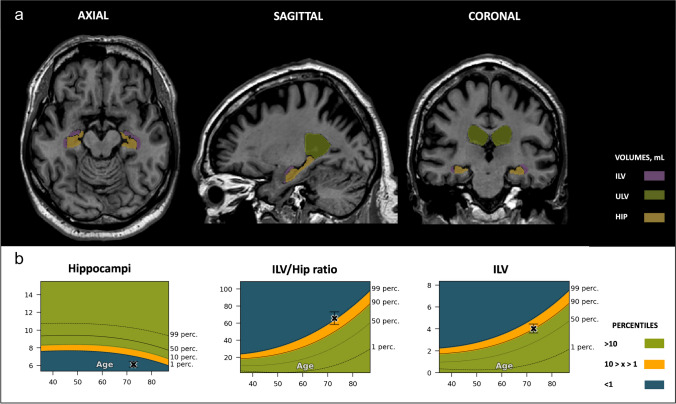
Mild cognitive impairment (MCI) case. Cross-sectional coronal T1-weighted image at the level of the medial temporal lobe and hippocampus with segmentation of the upper lateral ventricles (green), inferior lateral ventricle (purple), and hippocampus (yellow). **A** — Automated volumetric measurements *left*: axial view; *middle*: sagittal view; *right*: coronal view. **B** — Population graphs *left*: hippocampal volumes of this subject showing the gray “x” marker in the blue region, below the 1st percentile; *middle*: the ILV/Hip ratio of this subject showing the gray “x” marker in the blue region, just above the 99th percentile. *Right:* ILV volumes of this subject showing the gray “x” marker in the orange region, above the 90th percentile. Color-coded population graph percentile interpretation; 1–90th percentile, green (normal); 90–99th percentiles, orange (caution); > 99 th percentile; dark blue (abnormality)

The MCI patient’s (age, 72.7 years old) hippocampal brain structure segmentations fell below the 1st percentile range, indicating significant atrophy in this region (Fig. 6). Conversely, the ILV volume surpassed the 90th percentile, leading to an ILV/Hip ratio above the 99th percentile, signifying this outcome is rare among the healthy population. The visual MTA score for the MCI patient received a score of 2 for each of the measures, akin to the SCD subject.

**Fig. 7:**
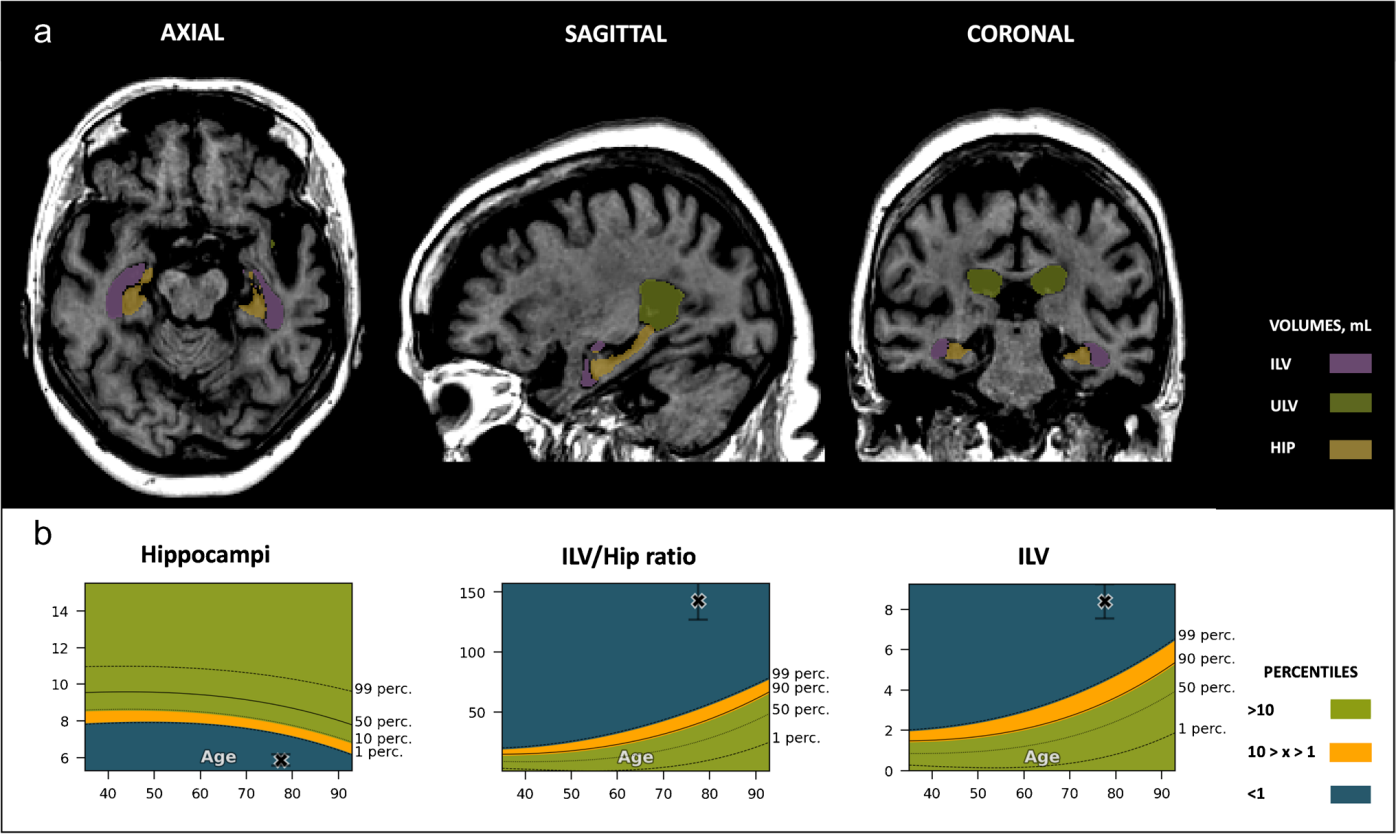
Dementia (DEM) case. Cross-sectional coronal T1-weighted image at the level of the medial temporal lobe and hippocampus with segmentation of the upper lateral ventricles (green), inferior lateral ventricle (purple), and hippocampus (yellow). **A** — Automated volumetric measurements *left*: axial view; *middle*: sagittal view; *right*: coronal view. **B** — Population graphs *left*: hippocampal volumes of this subject showing the gray “x” marker in the blue region, below the 1st percentile; *middle*: the ILV/Hip ratio of this subject showing the gray “x” marker in the blue region, above the 99th percentile. *Right*: ILV volumes of this subject showing the gray “x” marker in the blue region, above the 99th percentile. Color-coded population graph percentile interpretation; 1–90th percentiles, green (normal); 90–99th percentiles, orange (caution); > 99th percentile, dark blue (abnormality)

**Fig. 8:**
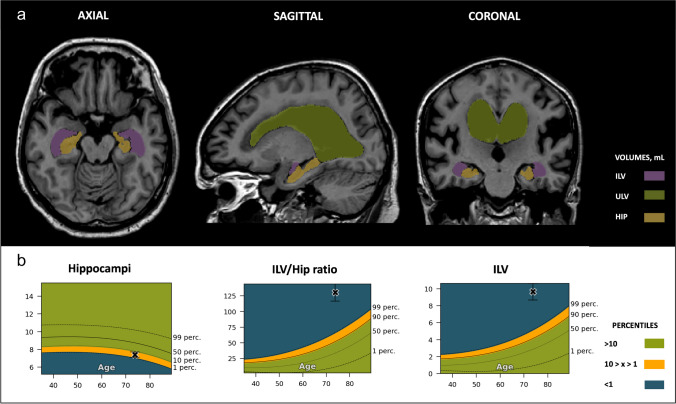
Normal-pressure hydrocephalus (NPH) case. Cross-sectional coronal T1-weighted image at the level of the medial temporal lobe and hippocampus with segmentation of the upper lateral ventricles (green), inferior lateral ventricle (purple), and hippocampus (yellow). **A** — Automated volumetric measurements *left*: axial view *middle*: sagittal view *right*: coronal view. **B** — Population graphs *left*: hippocampal volumes of this subject showing the gray “x” marker in the orange region, between the 1st and 10th percentile *middle***:** the ILV/Hip ratio of this subject showing the gray “x” marker in the blue region, above the 99th percentile. *Right*: ILV volumes of this subject showing the gray “x” marker in the blue region, above the 99th percentile. Color-coded population graph percentile interpretation; 1–90th percentiles, green (normal); 90–99th percentiles, orange (caution); > 99th percentile, dark blue (abnormality)

For the DEM patient (age, 77.5 years old), individual volumes, both ILV and HC, along with the corresponding ILV/Hip ratio, substantially exceeded the 99th percentile when compared to the healthy age-matched reference population (Fig. 7). Visual MTA assessment revealed a corresponding score of 4 for the left and 3 for the right hemisphere, culminating in a total consensus rating of 3.5. Lastly, in the case of NPH (age, 73.7 years old), despite having a normal hippocampal volume, there was a noticeable enlargement of the lateral ventricles (Fig. 8). This led to an ILV/Hip ratio that exceeded the 99th percentile, a characteristic observation in patients with normal pressure hydrocephalus, and one of the causes of dementia that can be managed and potentially reversed, with appropriate treatment. In alignment with the automated volumetric measurements, this patient received a consistent visual MTA score of 4, for both left and right hemispheres and the overall total MTA score.

## Discussion

In this study, an ILV/Hip ratio computed by icobrain dm as a potential additional metric in the diagnostic work up of neurodegenerative diseases such as AD was investigated. Our findings demonstrate that the performance of the ILV/Hip ratio score is, besides the aforementioned advantages of automated volumetry versus manual assessment, comparable to the results obtained by the consensus of three individual raters (with a high level of agreement in terms of inter-rater variability), indicating the ILV/Hip ratio score can serve as an additional (e.g., confirmative) metric for MTA atrophy stage and progression.

This study has shown that ILV volumes and ILV/Hip ratio scores show the highest correlation to visually assessed MTA ratings, in comparison to the automated hippocampal volumes versus the visually assessed MTA. This emphasizes the importance of not only considering the hippocampal volumes alone but also regarding the inferior lateral ventricle as an equally important structure in the MTA assessment in neurodegenerative disorders.

Regarding the ordinal logistic regression analysis, it needs to be noted that differences in coefficients among hippocampal volumes, ILV volumes, and the ILV/Hip ratio are influenced by variations in their scales and units. Specifically, the ILV/Hip ratio is a percentage, while individual hippocampal volumes and ILV volumes are measured in milliliters, making direct comparisons challenging. These differences emphasize the importance of relying on scale-invariant metrics, such as *AUC*, Brier scores, Kendall-Tau, and Spearman coefficients, for an accurate assessment of correlation strength. Despite the lower coefficients observed for the ILV/Hip ratio compared to the individual hippocampal and ILV volumes, the additional scale-invariant metrics reveal a stronger correlation to the visual MTA ratings. Lastly, regarding the correlation to cognitive outcome, the slightly lower correlation coefficients for individual ILV volumes suggest that, in contrast to visual MTA, the ILV/Hip ratio and hippocampal volumes, the individual ILV volumes may have, as was to be expected, a relatively less pronounced impact on cognitive function as assessed by MMSE scores. This observation, while anticipated, underscores the potential added value of incorporating the ILV/Hip ratio to enhance the precision of cognitive assessment beyond the singular focus on individual volumes. Furthermore, it needs to be noted that the MMSE, being a single time point and general measure of cognitive function that lacks the specificity needed for diagnostic differentiation, may not be as sensitive to early stages of cognitive decline. It might not capture subtle cognitive changes that occur in early phases of the disease, or individuals with near-normal (ceiling effect) or very low (floor effect) cognitive functions. In addition, different levels of education and diverse cultural backgrounds might introduce bias. Furthermore, the MMSE may not provide a comprehensive evaluation of all cognitive functions affected in AD.

In terms of diagnostic purposes, it needs to be stressed that the MTA score is not specific for AD, and normal imaging findings in the medial temporal lobe region does not exclude AD either, especially in the early stages. When assessing the classification accuracy, both the ILV/Hip ratio and the visual MTA ratings showed similar consistency in their ability to discriminate between different pairwise disease stage comparisons. The closely matched *AUC* values and overlapping *CI* values between different methods suggest no statistically significant difference in classification accuracy. These findings collectively show a robust and potentially equivalent predictive performance across the spectrum of disease severity, as evidenced by the improving discrimination metrics and decreasing prediction errors across the considered variables.

Moreover, considering their comparable specificity patterns, both total individual hippocampal volumes and the total ILV/Hip ratio are implicated in holding value for confirming specific diagnoses. Nevertheless, the consistently lower sensitivity (at the Youden index) observed in individual total hippocampal volumes suggests they may be less effective than the total ILV/Hip ratio in identifying borderline cases.

In the light of this study, the selection between employing the individual ILV volumes alone or the ILV/Hip ratio is contingent upon the specific clinical context and the prioritization of diagnostic considerations. Each approach presents distinct characteristics with implications for minimizing different types of diagnostic errors. Nonetheless, it needs to be noted that the ILV score alone will not be sufficient for adequate differential diagnosis in the presence of (additional co-) pathologies such as NPH, which needs to be considered for a correct interpretation of the ILV/Hip ratio score.

The ILV/Hip ratio showed a good balance between sensitivity and specificity for all pairwise comparisons (except SCD vs. CN), holding potential to reveal unique patterns that are not evident in the individual raw volumes. This can aid in identifying subgroup profiles within a cohort, which can have clinical implications (e.g., better clinical decision support), or be useful for patient stratification. Furthermore, the ratio provides a normalized measure that considers the relative ILV size compared to the hippocampal volume, which is particularly useful in comparing patients with varying brain sizes, improving diagnostic consistency and comparability.

While the ILV/Hip ratio may not consistently outperform other measurements in terms of classification accuracy, it can be a useful complementary quantitative tool, particularly in certain diagnostic contexts, for example when examining the trade-off between sensitivity and specificity.

To determine the applicability of the automated volumetric segmentations such as the ILV/Hip ratio score in routine clinical practice, it is essential to be able to distinguish between what is considered part of a healthy aging pattern and what is not. The existing guidelines for visual MTA scoring are well-established and include a widely accepted age-related cut-off at 75 years old (with an MTA score of 1.5 or more in both hemispheres considered “abnormal” in younger patients (age < 75) and an MTA score of 2 or more in both hemispheres being abnormal at age > 75) [33, 48]. However, the optimal coronal slice position for MTA scoring has not been universally agreed upon, lacking a definitive consensus or established criteria thereof [37]. This might lead to inconsistency and scoring variations, which stands in contrast to automated tools that consistently rely on a predefined coronal slice position. Thus, the ILV/Hip ratio score yielding a continuous variable, in contrast to the visual MTA score employing a scale ranging from 0 to 4, allows for a more standardized and fine-grained determination of normality and abnormality, which, when combined with population graphs, holds potential clinical relevance.

The ability to interpret ILV/Hip ratio score measurements using age and sex-correlated population graphs is an enrichment to the already continuous characteristic of the ILV/Hip ratio score. A continuous measurement would enable close monitoring of gradual decline, which prevents a situation where a patient aged 74, with an MTA score of 1.5 (pathological in this case) can have a normal MTA score in a follow-up examination the year after, solely due to the surpassing of the aforementioned (stringent) threshold, since subtle changes and trends are not depicted in a visual assessment. In addition, it needs to be noted that the existence and inconsistency in the use of different (age-specific and clinical population-based) cut-offs for defining visual MTA rating scale abnormalities is identified as a major issue and source of heterogeneity, with only a few studies addressing this concern so far [24]. Thus, preserving the advantage of automated volumetric imaging quantification, the ILV/Hip ratio score can be translated to a standardized value useful for interpretation purposes when compared to an age- and sex-matched normative population, while retaining a comparable performance to visual assessment.

In fact, numerous studies confirm the superiority of automated volumetry over visual rating [3, 4, 6, 8, 22, 34, 49], strengthening the potential beneficial use of automated visual rating scales in routine clinical practice. However, it is important to consider the existing inter-software variability in automated volumetry, which can give rise to differing clinical interpretations, emphasizing the need to avoid assuming interchangeability of software applications [54]. Although not explored in this study, another potential limitation of the ILV/Hip ratio score versus visual MTA assessment is the introduction of scanner specific dependency, an undesired effect. Using a diverse dataset in training deep learning-based image segmentation methods, as in icobrain v 5.10, has been shown to lead to lower inter-scanner variability [28]. Furthermore, the performance in terms of reproducibility of the ILV/Hip ratio was not assessed in this paper. However, software reproducibility has been previously described and validated by Wittens and Allemeersch et. al, (2021) [52]. Besides age and gender, education can also be seen as a confounder in MTA grading, which was not taken into account in the current study and should be part of further validation studies [50]. An additional challenge in this study is the usage of a convenience sample, where not all diagnoses were substantiated by CSF biomarkers. While this does not affect the inter-observer findings, it does introduce complexity in accurately interpreting abnormalities. The NI-AAA criteria were applied wherever possible; however, due to the retrospective nature of the study, coupled to common constraints in routine clinical practice, including contraindications for lumbar puncture (e.g., coagulation disorders, thrombocytopenia, use of anticoagulants, increased intracranial pressure, and resilience against or incapacity to participate in the procedure), not all criteria for each subject were met in full to obtain a biomarker-based diagnosis. Lastly, it needs to be noted that the consideration of the visual MTA as a suboptimal golden standard in the context of automated volumetry may not necessarily directly lead to substantial improvements in the field. However, since the visual MTA is often used as a reference point for diagnosis and treatment decisions, validating against this standard ensures that automated methods align with established clinical practices, making them more readily applicable and interpretable in real-world scenarios. Showing equivalence of alternative or complementary automated methods with the familiarity, simplicity, and ease of implementation that the visual MTA rating provides to clinicians can aid in gaining acceptance and establishing trust in the reliability and accuracy of automated methods, justifying their integration into routine practice [12].

In future studies, validation on larger clinical datasets containing MRI acquisitions of different scanner types to evaluate the generalizability of the ILV/Hip ratio score, as well as further fine tuning the percentiles as alternative interpretable “thresholds” for the ILV/Hip ratio score that correspond to specific visual MTA rating scores, are needed to determine, among others, the diagnostic utility. Lastly, it would be valuable to investigate the combined use of an automated equivalent of the entorhinal cortex atrophy score (ERICA) [10], which utilizes the entorhinal cortex, parahippocampal gyri, and amygdala as primary structures for assessing atrophy patterns and the automated MTA alternative to further improve the accuracy and specificity of neurodegenerative disease diagnosis and monitoring.

## Conclusion

The ILV/Hip ratio score showed an excellent correlation to the visually assessed MTA consensus rating, currently regarded as the golden standard for MTA scoring. The less strong correlation of this visually assessed MTA consensus rating to hippocampal volumes, which has become a widely accepted additional informative metric in MTA assessment, emphasizes the potential use of the ILV/Hip ratio score in a heterogeneous patient population. Furthermore, the possibility to calibrate the ILV/Hip ratio using age- and sex-matched healthy population graphs has an added value for future research to validate the use of automated volumetry.

### Supplementary information

Below is the link to the electronic supplementary material.

Supplementary file1 (DOCX 38 KB)
