# Supplementary material for: Towards validation in clinical routine: a comparative analysis of visual MTA ratings versus the automated ratio between inferior lateral ventricle and hippocampal volumes in Alzheimer’s disease diagnosis
Source: Neuroradiology. 2024 Jan 19;66(4):487–506. doi: 10.1007/s00234-024-03280-8 (PMC10937807; doi:10.1007/s00234-024-03280-8)
Supplement: Supplementary file 1 — Supplementary file1 (DOCX 38 KB) [file 234_2024_3280_MOESM1_ESM.docx]

**Supplementary material Table 1:** 3D T1 sequence scan parameters.

| **Sequence** | TFE |
| --- | --- |
| **Coil** | 32 channels |
| **Field of view (mm³)** | 230 x 230 x 172.8 |
| **Phase oversampling** | 12% |
| **Acq. resolution (mm³)** | 1.2 x 1.2 x 1.2 |
| **Reco. resolution (mm³)** | 0.6 x 0.6 x 0.6 |
| **Slice orientation** | sagittal |
| **TE (ms)** | 2.8 |
| **TR (ms)** | 7.6 |
| **TI (ms)** | 800 |
| **bandwidth (Hz/pixel)** | 309.6 |
| **TFE factor/ETL** | 144 |
| **Flip angle** | 8° |
| **Acceleration technique** | compressed sense |
| **Acceleration factor** | 2 |
| **Scan time** | 2 min 44 s |

**TFE**=Turbo Field Echo, **ETL**=Echo Train Length.

**Supplementary material Table 2:** Healthy reference population datasets.

| **Name** | **#** | **Age range** | **Scanner and field strength (T)** | **Source** |
| --- | --- | --- | --- | --- |
| **IXI** | 600 | 20-86 | Philips Gyroscan Intera, 1.5T  Philips Intera, 3T  GE Medical Systems. 1.5T | <http://brain-development.org/ixi-dataset/> |
| **OASIS_NC_CROSS** | 316 | 18-96 | Siemens Vision, 1.5T | <http://www.oasis-brains.org/> |
| **NKI** | 406 | 6-85 | Siemens TrioTim, 3T | [http://fcon_1000.projects.nitrc.org/indi/enhanced/](http://fcon_1000.projects.nitrc.org/indi/enhanced/d) |
| **MMRR-Kirby 21** | 21 | 22-61 | Philips ACHIEVA, 3T | <http://www.nitrc.org/frs/?group_id=313> |
| **MIRIAD_NC** | 23 | NA | GE Medical Systems Signa, 1.5T | NeuroImage 2013, 70:33-36 |
| **OASIS_NC_LONG** | 38 | 60-96 | Siemens, 1.5T | <https://www.ncbi.nlm.nih.gov/pubmed/19929323> |
| **Brainomics** | 94 | 18-49 | Brucker, 3T  Siemens TrioTim, 3T | <https://doi.org/10/1016/j.neuroimage.2015.09.052> |
| **OPEN Neuro** | 120 | 18-31 | Siemens TrioTim, 3T | <https://openneuro.org/datasets/ds000243/versions/00001> |
| **OPEN Neuro** | 60 | 18-30 | Siemens SKYRA, 3T | <https://openneuro.org/datasets/ds000053/versions/00001> |
| **OPEN Neuro** | 21 | 21-50 | Siemens ALLEGRA, 3T | <https://openneuro.org/dataset/ds000101/versions/00004> |
| **OPEN Neuro** | 63 | 21-77 | Siemens TrioTim, 3T | <https://openneuro.org/datasets/ds000240/versions/00002> |
| **OPEN Neuro** | 95 | 18-30 | Philips ACHIEVA, 3T | <https://openneuro.org/datasets/ds000202/versions/00001> |
| **OPEN Neuro** | 26 | 19-50 | Siemens ALLEGRA, 3T | <https://openneuro.org/datasets/ds000102/versions/00001> |
| **Studyforrest** | 20 | 20-35 | Philips ACHIEVA, 3T | <http://studyforrest.org/access.html> |

**Supplementary material Table 3:** Population graph performance specifications of each brain structure volume/ratio. **ILV =** Inferior lateral ventricle. **Hip** = Hippocampus. **EB =** Error bar**.**

| **Variable** | **EB** |
| --- | --- |
| Hippocampus (mL) - Total | 0.4 |
| Hippocampus (mL) - Left | 0.2 |
| Hippocampus (mL) - Right | 0.2 |
| ILV (mL) - Total | 0.2 |
| ILV (mL) - Left | 0.1 |
| ILV (mL) - Right | 0.1 |
| ILV/Hip ratio (%) - Total | 2.2 |
| ILV/Hip ratio (%) - Left | 2.5 |
| ILV/Hip ratio (%) - Right | 2.4 |

**Supplementary material Table 4:** Percentiles and MTA consensus scores per subject.

| **Subjects** | **Clinical Diagnosis** | **MTA, Left** | **MTA, Right** | **MTA, Total** | **ILV/Hip, Left** | **ILV/Hip, Right** | **ILV/Hip, Total** |
| --- | --- | --- | --- | --- | --- | --- | --- |
| 1 | CN | 0 | 0 | 0 | 67 | 78,84061905 | 73,4726839 |
| 2 | CN | 0 | 0 | 0 | 85 | 60 | 77 |
| 3 | CN | 0 | 0 | 0 | 27,1307768 | 59,99403566 | 41,52849194 |
| 4 | CN | 1 | 0 | 0,5 | 100 | 65,83045593 | 98,00439045 |
| 5 | CN | 1 | 1 | 1 | 82,69816326 | 92,92923322 | 89,79860003 |
| 6 | CN | 0 | 0 | 0 | 71,93439764 | 7,991010985 | 38,0064363 |
| 7 | CN | 0 | 0 | 0 | 30 | 16,05707379 | 24,00820766 |
| 8 | CN | 0 | 0 | 0 | 20,24743425 | 20,32470916 | 17,10235903 |
| 9 | CN | 1 | 1 | 1 | 57,51718712 | 91,46037257 | 78,73705867 |
| 10 | CN | 0 | 0 | 0 | 86 | 70,8165709 | 81,16481082 |
| 11 | CN | 1 | 1 | 1 | 98,50388939 | 100 | 100 |
| 12 | CN | 0 | 0 | 0 | 29,2495396 | 81,72649311 | 59,5538395 |
| 13 | CN | 0 | 0 | 0 | 83 | 81,81297996 | 82 |
| 14 | CN | 2 | 2 | 2 | 96,12757352 | 100 | 98,97205617 |
| 15 | CN | 0 | 0 | 0 | 69 | 22,44419958 | 47,39111311 |
| 16 | CN | 0 | 0 | 0 | 76,4800867 | 46,90515428 | 68,9147405 |
| 17 | SCD | 0 | 0 | 0 | 27,19168984 | 53 | 38,35246388 |
| 18 | SCD | 0 | 0 | 0 | 39,59530502 | 4,112626568 | 18,7062122 |
| 19 | SCD | 0 | 0 | 0 | 57,58048626 | 53 | 56,62821223 |
| 20 | SCD | 0 | 0 | 0 | 52,42900257 | 20,14434656 | 41,79751653 |
| 21 | SCD | 0 | 1 | 0,5 | 69,13622291 | 90,93909286 | 83,36663871 |
| 22 | SCD | 0 | 0 | 0 | 95,19274832 | 64,04871181 | 87,69318037 |
| 23 | SCD | 0 | 0 | 0 | 11,88534038 | 9,744198615 | 8,756064297 |
| 24 | SCD | 1 | 2 | 1,5 | 100 | 100 | 100 |
| 25 | SCD | 1 | 1 | 1 | 68 | 60,5058679 | 66 |
| 26 | SCD | 1 | 1 | 1 | 17,57365569 | 0,615537374 | 3,96764887 |
| 27 | SCD | 0 | 0 | 0 | 68 | 47,08948333 | 60 |
| 28 | SCD | 2 | 1 | 1,5 | 97,55333283 | 86,96498988 | 94,71616598 |
| 29 | SCD | 0 | 0 | 0 | 35,48224886 | 14,26183259 | 26,66939739 |
| 30 | SCD | 0 | 0 | 0 | 29,10410568 | 16,64619514 | 23,81125698 |
| 31 | SCD | 0 | 0 | 0 | 28,56295328 | 25,82101055 | 21,97084628 |
| 32 | SCD | 0 | 0 | 0 | 72 | 29,93267531 | 53,29667488 |
| 33 | SCD | 0 | 0 | 0 | 19,47165488 | 38,84009946 | 25,7241538 |
| 34 | SCD | 1 | 1 | 1 | 14,33061118 | 24,48517517 | 15,37502869 |
| 35 | SCD | 2 | 1 | 1,5 | 88,71345437 | 76,76713283 | 85,77297837 |
| 36 | SCD | 0 | 0 | 0 | 69,21370457 | 46,60508098 | 58,71050105 |
| 37 | SCD | 0 | 0 | 0 | 47,67089377 | 50,47162925 | 45,04244026 |
| 38 | SCD | 2 | 2 | 2 | 100 | 97,59362852 | 99,72644406 |
| 39 | SCD | 1 | 1 | 1 | 95,18818442 | 85,03468293 | 92,89078088 |
| 40 | SCD | 2 | 1 | 1,5 | 97,77315387 | 95,88593328 | 97,58583895 |
| 41 | SCD | 1 | 1 | 1 | 73,70266705 | 52,00188003 | 66 |
| 42 | SCD | 1 | 1 | 1 | 91,61994227 | 52,5058894 | 83,01273902 |
| 43 | SCD | 0 | 0 | 0 | 71,61409984 | 17,29367944 | 46,98689374 |
| 44 | SCD | 1 | 2 | 1,5 | 94,79057693 | 100 | 99,4680277 |
| 45 | SCD | 0 | 0 | 0 | 64 | 20,06077588 | 44,4173459 |
| 46 | SCD | 1 | 2 | 1,5 | 60,89462375 | 58,79937851 | 62,24156824 |
| 47 | SCD | 1 | 1 | 1 | 74 | 52,6037637 | 66 |
| 48 | SCD | 2 | 1 | 1,5 | 98,26425167 | 80,54156669 | 93,2656128 |
| 49 | SCD | 2 | 2 | 2 | 90,40552601 | 96,67699875 | 95,37225763 |
| 50 | MCI | 2 | 2 | 2 | 99,68856508 | 100 | 100 |
| 51 | MCI | 2 | 2 | 2 | 68,47463168 | 94,88330845 | 86,81519513 |
| 52 | MCI | 2 | 2 | 2 | 99,52913303 | 94,23196032 | 99,10915104 |
| 53 | MCI | 0 | 0 | 0 | 80,36309216 | 20 | 60,187503 |
| 54 | MCI | 1 | 1 | 1 | 98,9966161 | 99,68685646 | 100 |
| 55 | MCI | 0 | 0 | 0 | 31,17936419 | 31,00182447 | 30 |
| 56 | MCI | 1 | 1 | 1 | 37,39252692 | 55,79605431 | 44,32740434 |
| 57 | MCI | 1 | 1 | 1 | 22,4110442 | 25,08344325 | 20,8760439 |
| 58 | MCI | 1 | 1 | 1 | 79,39029942 | 92,27254018 | 87,49076916 |
| 59 | MCI | 1 | 2 | 1,5 | 62,47017139 | 91,87196089 | 78,31550625 |
| 60 | MCI | 1 | 1 | 1 | 22,03764731 | 26,13356702 | 21,02717275 |
| 61 | MCI | 2 | 2 | 2 | 100 | 100 | 100 |
| 62 | MCI | 2 | 2 | 2 | 72,3865868 | 48,95865887 | 57,52478959 |
| 63 | MCI | 3 | 3 | 3 | 100 | 100 | 100 |
| 64 | MCI | 2 | 2 | 2 | 99,38929259 | 100 | 99,68559883 |
| 65 | MCI | 0 | 0 | 0 | 38,33116144 | 2,682756254 | 14,68630085 |
| 66 | MCI | 1 | 1 | 1 | 92,66838908 | 98,2356974 | 96,13133944 |
| 67 | MCI | 1 | 1 | 1 | 56,54643322 | 69,71700667 | 64,22323771 |
| 68 | MCI | 2 | 2 | 2 | 95,49030268 | 100 | 99,32424939 |
| 69 | MCI | 1 | 1 | 1 | 38 | 38 | 36,83787815 |
| 70 | MCI | 1 | 0 | 0,5 | 36,66842811 | 55,65287876 | 45,55855156 |
| 71 | MCI | 2 | 1 | 1,5 | 98,33175528 | 94,37446173 | 97,09795627 |
| 72 | MCI | 1 | 1 | 1 | 27,35542674 | 70,70294747 | 47,19491808 |
| 73 | MCI | 1 | 1 | 1 | 47,55738651 | 23,18759929 | 39,76291435 |
| 74 | MCI | 3 | 2 | 2,5 | 68,59894138 | 65,20630092 | 65,18722169 |
| 75 | MCI | 2 | 2 | 2 | 97,55100918 | 96,88339005 | 98,01755632 |
| 76 | MCI | 1 | 1 | 1 | 74,89159418 | 84,89415096 | 78,0238392 |
| 77 | MCI | 2 | 3 | 2,5 | 100 | 100 | 100 |
| 78 | MCI | 0 | 0 | 0 | 24,57605103 | 4,834052337 | 13,48501225 |
| 79 | MCI | 2 | 2 | 2 | 100 | 100 | 100 |
| 80 | MCI | 0 | 0 | 0 | 77 | 59,01363639 | 70 |
| 81 | MCI | 0 | 0 | 0 | 49,8066651 | 25,77239401 | 40,10785337 |
| 82 | MCI | 1 | 1 | 1 | 75,56702465 | 67,79168994 | 74,65818203 |
| 83 | MCI | 2 | 2 | 2 | 99,04108867 | 99,21339703 | 99,59043195 |
| 84 | MCI | 2 | 1 | 1,5 | 42,12139539 | 85,172174 | 65,48109251 |
| 85 | DEMENTIA | 1 | 1 | 1 | 23,33330443 | 79,4922263 | 40,81693968 |
| 86 | DEMENTIA | 1 | 2 | 1,5 | 88,8009843 | 98,8236172 | 96,90294075 |
| 87 | DEMENTIA | 2 | 3 | 2,5 | 100 | 100 | 100 |
| 88 | DEMENTIA | 1 | 1 | 1 | 97,45051508 | 68,59488749 | 88,26909301 |
| 89 | DEMENTIA | 3 | 3 | 3 | 100 | 100 | 100 |
| 90 | DEMENTIA | 2 | 3 | 2,5 | 99,87926489 | 100 | 100 |
| 91 | DEMENTIA | 2 | 2 | 2 | 97,57469601 | 99,02709915 | 99,22485111 |
| 92 | DEMENTIA | 2 | 3 | 2,5 | 92,74626625 | 100 | 100 |
| 93 | DEMENTIA | 2 | 2 | 2 | 100 | 95,50748933 | 99,2091528 |
| 94 | DEMENTIA | 3 | 2 | 2,5 | 100 | 98,19154798 | 99,84566303 |
| 95 | DEMENTIA | 4 | 3 | 3,5 | 100 | 100 | 100 |
| 96 | DEMENTIA | 1 | 1 | 1 | 72,5652663 | 99,34449295 | 95,49910661 |
| 97 | DEMENTIA | 1 | 1 | 1 | 100 | 97,39702215 | 100 |
| 98 | DEMENTIA | 1 | 0 | 0,5 | 96,54977686 | 73,37369377 | 90,80811985 |
| 99 | DEMENTIA | 3 | 3 | 3 | 100 | 100 | 100 |
| 100 | DEMENTIA | 3 | 3 | 3 | 100 | 100 | 100 |
| 101 | DEMENTIA | 4 | 2 | 3 | 100 | 99,06272792 | 100 |
| 102 | DEMENTIA | 3 | 2 | 2,5 | 100 | 98,01898961 | 100 |
| 103 | DEMENTIA | 3 | 3 | 3 | 100 | 100 | 100 |
| 104 | DEMENTIA | 3 | 3 | 3 | 100 | 100 | 100 |
| 105 | DEMENTIA | 2 | 2 | 2 | 72,17290992 | 99,4076389 | 94,52777761 |
| 106 | DEMENTIA | 2 | 2 | 2 | 68,03890365 | 96,44952243 | 87,97400986 |
| 107 | DEMENTIA | 2 | 4 | 3 | 100 | 100 | 100 |
| 108 | DEMENTIA | 3 | 2 | 2,5 | 100 | 100 | 100 |
| 109 | DEMENTIA | 1 | 1 | 1 | 86,88796524 | 99,41786359 | 97,79083395 |
| 110 | DEMENTIA | 2 | 2 | 2 | 84,26732916 | 99,28552345 | 97,09064284 |
| 111 | DEMENTIA | 4 | 2 | 3 | 100 | 57,44367348 | 99,03142703 |
| 112 | NPH | 4 | 4 | 4 | 100 | 100 | 100 |

**CN** = Cognitively healthy controls. **SCD** = Subjective cognitive decline subjects. **MCI** = Mild cognitive impairment patients. **DEM** = Dementia. **NPH** = Normal pressure hydrocephalus. **MTA** = Medial temporal lobe atrophy; **ILV** = Inferior lateral ventricle. **Hip** = Hippocampus.
